# Cremation

**Published:** 1881-09

**Authors:** C. W. Purdy

**Affiliations:** Chicago


### ARTICLE VI.

CREMATION. By Dr. C. W. PURDY, of Chicago. (Concluded from page 200, Aug. No., 1881.)

In 1850, a Report on a General Scheme for Extra-mural Sepulture was presented to both houses of Parliament, by Carlisle, Ashley, Chadwick and Smith. The report contains an account of a large number of London church-yards, showing their bad condition and the remarkable prevalence and virulence of cholera in their vicinity. Dr. Sutherland testified "that he had witnessed several outbreaks of cholera in the vicinity of grave-yards, which left no doubt on his mind as to the connection between the disease and such local influences; and that it is only when some epidemic comes that the consequence of long antecedent neglect becomes so apparent as to direct attention and excite alarm."

Burial grounds are unquestionably ruinous to health, as both theory and facts amply demonstrate; many sections of population suffer annually diseases and death, which are exposed to their influences; all engaged in this unwholesome system suffer—the grave-diggers, the gardeners, the men who repair the vaults and tomb-stones, the friends who visit the graves, and the whole funeral procession are exposed directly. There is no redeeming feature about this burial system, degrading to the dead and dangerous to the living.

Under the general adoption of cremation of our dead all this would be changed. The body would be quickly, cheaply and harmlessly rendered back to atmosphere and earth; to the former, carbonic acid and watery vapor; to the latter, ammonia and earth salts;—nothing is left corrupt, but rapidly cleansed and purified. Instead of the damp, cold shroud of a decaying grave, shared by worms and reptiles the most loathsome, the atmosphere of light would enshroud the etherialized remains in the brightness and purity of living day.

There is one feature of the burial system which we have but time to notice briefly in passing,—the most shocking to our feelings and dreadful to the imagination. We refer to the awful possibilities of living burial. This has happened, and it will happen. Hardly a grave-yard is opened but coffins are found containing bodies not only turned, but skeletons contorted in the last, hopeless struggle for life underground.

The *London Times*, May, 1874, says: "In August of 1873, a young lady died soon after her marriage. Owing to the intense heat she was buried the same day. Within a year her husband married again, and the mother of his first bride resolved to move her daughter's body to her native town. They open the vault, and find the poor girl's body fallen prostrate, her hair dishevelled, her shroud torn in pieces. She had risen, and burst her narrow prison to find herself in one a little larger, but far more inexorable. How long did she cry piteously for help? How long did she struggle and pray? We cannot tell: we can but guess her agonies!"

Much might be written on the subject of contamination of springs in the neighborhood of grave-yards, and there is no limit to the poisoning of such waters. One author states that there is not a pure stream of water throughout the whole of England. Is it any wonder then that that country is such a hot-bed of typhoid fever.

A fearful epidemic decimated the villages of Rotondella and Bollita, which was clearly traced to the water which had been drained into the wells through the neighboring hill-cemetery. Hundreds of wells have been found tainted so badly from the same cause as to necessitate their disuse. At Paris, M. Duchamp discovered a spring that filtered entirely through cemeteries and tasted strongly.

Dr. Pappenheim declares that springs tainted with organic matter of grave-yards are often found at great distances from their sources of pollution. But there is no necessity of multiplying instances of a fact that has come under the notice of the most of us.

Let us turn from the dark and gloomy picture of inhumation, whose perpetuated shadows darken the hours of so many happy homes, to the brighter, because purer, more beautiful, because more human, system of cremation.

Of late years a tide of opinion seems to be setting in in favor of the old custom. For the last ten years many distinguished physicians and chemists of Italy have warmly advocated the general adoption of cremation. In 1874, a congress was called to consider the matter at Milan, and petitioned the Chamber of Deputies for a clause in the sanitary code, permitting cremation under supervision of the syndices of the code, which was granted, and the result has been so far successful, that about seventy bodies were cremated in Milan in 1880.

In Switzerland, Dr. Ercolani is the champion of the cause, and there are several associations for its support. So long ago as

1797, cremation was seriously discussed by the French Assembly, and the events of the Franco-Prussian war have again brought the subject under notice of the medical press. Vienna has made cremation permissible, and cremation is practiced in the Austrian Capital. Dresden, Leipsic and Berlin are the centers of the German movement, which is quite as progressive in the matter as Italy.

In England, Dr. Lord, for many years medical health officer for Hampstead, continued to urge the practical necessity for the introduction of the system. Sir Henry Thompson, however, first brought the question prominently before the public, and started in 1874 the Cremation Society of London, the objects of which were stated to be to introduce through the agency of cemetery companies, municipal authorities and burial boards, some rapid process of disposing of the dead, which could not offend the living and should render the remains innocuous."

Thompson used one of Siemen's furnaces, consisting of—

1. A generator, in which the coal or wood generating the gas mixed in certain proportion with the air.

2. A regenerator or square chamber, intersected with perforated brick walls, which receives the gases at a high temperature.

3. A calefactor or actual combustion room ; this chamber is flooded with a gas and air at a temperature of 2000° F. It contains a cylindrical vessel about seven feet long, by five feet six inches in diameter; into that vessel is deposited the body, the whole at a white heat.

The gases of combustion rush off but are not allowed to taint the atmosphere; they are caught and carried over irregularly disposed layers of heated bricks till oxidation is complete. Not a particle of smoke at length arises from the chimney, and nothing but a little harmless gas reaches the outer air; throughout nothing repulsive is smelt or seen.

Prof. Gorini estimates the cost of cremating an ordinary body at from $10 to $15. The expense attendant on reducing a body in the furnace of Dr. Le Moyne, at Washington, Pa., is $15. The actual cost of incineration in the Siemens regenerating furnace is only 75 cents. These estimates are for single bodies, which would become very much reduced by the general adoption of the system, as will appear obvious.

The first body that was cremated in the United States, according to Dr. Le Moyne, was Colonel Henry Laurens, a member of the military family of General Washington. His body was cremated in South Carolina, in 1796. The second was that of Henry Barry, who lived and was cremated in the vicinity of Marion, South Carolina. The third was the body of Baron de Palm, cremated in the furnace erected by Dr. F. J. Le Moyne, in Washington, Pa., December, 1876. The fourth was that of Dr. Winslow, of California, cremated in Salt Lake City, in a temporary furnace erected through his request by the administrators of his estate. The fifth was the child of Julius Kircher, in his furnace in New York City, in the autumn of 1877.

During the first two years after the erection of the Washington Crematory nearly a hundred applications were made for cremation, which were declined, for the reason, as Dr. Le Moyne says, "that it was not constructed to be operated as a business, and an occasional cremation only was permitted for the purpose of keeping the subject before the public eye." I am informed however, by Mr. J. V. Le Moyne, that, since his father's death, the executors of the estate have thrown open the crematory for the use of those who desired to be cremated at the cost of incineration, and accordingly some sixteen bodies have been cremated in the Washington Crematory during the past year.

The process of disposing of the dead by cremation, as proposed and recently advocated, is not destruction by fire, or burning in the sense that many suppose. The body is reduced to ashes by the chemical action of intense heat, at a temperature of 2,000 to 3,000 degrees, without contact with flame or fire.

As an illustration we quote from one of the daily papers, observations taken at the cremation of the wife of a German physician in Dresden, November 6, 1874: "The hall around the furnace was decorated with flowers, and in every other respect the solemnity which should attend so serious a site was duly observed. The process of cremation was screened from the eyes of the lady's friends by an iron door. There was no smoke nor any unsightly transformation of the body. When the coffin was consumed, the body appeared in its natural state, then red-hot, and at last appeared to be of translucent white. From this it crumbled into ashes. Up to the period of its entire consumption, the process was merely a rapid drying up."

In the ordinary Siemen's Regenerating Furnace (used by Reclam in Germany, and Thompson in England), only hot blast is used, the body supplying the hydrogen and carbon, or a stream of heated hydro-carbon, mixed with heated air, is sent from a gasometer supplied with coal or wood, the chamber being heated to a high degree before cremation begins. The advantages of this furnace are, that the heat of the expended fire is nearly all retained by the regenerator, and the gas retort admits of production being stopped without much loss. The noxious gases which are produced during the first five minutes or so of combustion, pass through a flue into a second furnace and are entirely consumed. Bodies may be reduced in this furnace in an hour or less to from three to six pounds of pure white ash, or lime dust. Thompson reduced a body weighing 144 pounds in fifty minutes to about four pounds of ash.

Hawes describes the cremation ceremony, as proposed, in the following language: "The body is conveyed to the crematory and has been placed in its shroud, or, if you will, even in the coffin, at the mouth of what seems to be a small cave at the entrance of a Gothic edifice; the door is closed, and whilst the mourners enter the funeral chapel to hear the service read, the body glides into the crematory by the simplest machinery, and is returned in the words of your burial service: "Dust to dust, ASHES to ASHES," words culled from the old Greek or Roman cremation ritual, misapplied for centuries to Christian burial." No slowly putrefying form remains in the damp, cold ground, with its dreaded memories to haunt the imagination and sting the sensibilities of the living.

Cremation seeks not, as many suppose, to divorce the revered memories or tender affections from the honored dead; but rather it would rob the last resting-place of "the small cold worm that fretteth the enshrouded form." It would separate from the dead only those memories which fill the breast with horror, and feed the imagination with dreadful forebodings of the corruptible before it "put on incorruptible."

Plant still if you will in the sublimated ashes of the departed, the myrtle and the ivy, the primrose and the laurel, whose budding foliage shall cluster about the golden urn and form a perfumed veil of beauty, at once an emblem of the purified remains and a beautiful allegory of the hereafter.

It has been argued against cremation that it interferes with the doctrine of the Resurrection. If this were true, what becomes of the army of martyrs who have perished by fire, and many thousands of good and worthy people who have been burned in conflagration ? Did not David thank the people of Jabesh-Gilead for burning Jonathan; nay, did not God himself command Abraham to offer up his son Isaac on the altar, and though he stayed his hand, requiring not the sacrifice, yet the funeral pile and its contingencies were fully authorized by God himself.

But granted even the old doctrine of a literal resurrection, and cremation does not affect it, for a power capable of restoring and refashioning atoms dispersed through the metamorphosis of centuries, would be equally capable of restoring the atoms dispersed by fire in a brief hour.

A more reasonable objection has been urged by opponents of cremation, on the grounds of depriving the courts of means which often convict criminals in poisoning cases. The many great advantages of adopting cremation as a general practice are so weighty as vastly to counterbalance these. Indeed, we have not in man's history any great benefit resulting from a system or practice, but it is attended by its consequent minor evils ; no great public good but has its attendant draw-backs. Who, for instance, would prohibit public festivals and holidays, because theft, debt and drunkenness are increased thereby ? Who would stop the sale of weapons to prevent suicides ; yet the advantages claimed for cremation have greater demands on public policy and safety than most of these.

Not the least objection to the present system of inhumation is its enormous attendant expense, a tax so great on the resources of many poor families, that it actually amounts to oppression. The savings of months or years, which perhaps was the only reliance against temporary reverses or actual want; and perhaps might have kept starvation from the door, is swept away by the almost absolute necessity of keeping up the appearances of what are termed a decent Christian burial. We have no hesitation in saying that the vast sums of money spent on funerals would keep every needy family in the land from hunger and want. The practice of general cremation would dispose of the dead more in keeping with the laws of nature; more safely to the dead and to the living; in short, more desirably in all respects at a cost less than one-fifteenth the sum now squandered to embellish the earth as a dead-house.

Figures are strong facts. It costs Chicago $1,000,000 annually for funerals alone, and it cost Illinois, in 1880, $6,000,-000 for the same purpose, which is nearly one-third the amount of internal revenue yielded by the State for the same period. It costs the United States over $100,000,000 annually for funerals, and the saving which would result from generally adopting cremation would amount to a sum sufficient to pay off our national debt in about fifteen years; while our government has not been able, with the immense revenue yielded by this country, to reduce one-third of this debt in the same length of time. One and one-fourth times more money is spent annually on funerals in the United States than the government expends for public school purposes. Funerals cost two and a half times more money annually than would buy the grounds, buildings and apparatus of all the universities and colleges in this country. The amount of money expended on funerals in the United States, if saved for a fraction over four years, would equal the total sum disbursed by our government for pensions during the last twenty years.

Funerals cost this country in 1880 enough money to pay the liabilities of all the commercial failures in the United States in the same year, and give each bankrupt a capital of $8,630 to begin business again. Funerals cost annually more money than the value of the combined gold and silver yield of the United States in the year 1880. The population of the Christian world, in round numbers, is about 400,000,000 of people, or a little less than one-third the entire population of the earth. At the average cost of burying the dead in the United States the Christian world would spend annually for funerals $800,000,000 at a safe estimate. Let this annual expenditure of money be saved, with its accrued interest at six per cent., for little over twenty years, and it would pay off the combined national debt of the world.

What a vast amount of want and misery the judicious expenditure of this money would save the human race! It would wipe out pauperism from civilization. Yet this does not by any means express the gigantic expense of the burial system, for, remember, we have only now spoken of funerals. Add yet to these the sums of money invested in the millions of acres of valuable property used for burial-grounds; the immense sums lavished in erecting costly monuments and embellishing graves; the depreciation in the value of residence property in the vicinity of these grave-yards. Add these together, and we have a sum expended in maintaining these cities of the dead so stupendous, that it is well calculated to arrest the attention of those whose business it is to deal with questions of public policy.

And to what useful purpose to the dead, or the living, is this vast fund so recklessly—I would say, criminally—squandered? To the former, to preserve, against nature's laws, the putrid relics of organic man, in his most humiliating and degraded condition, long years after nature's laws would have transformed him into many varied and lovely forms of renewed organic life, surrounded by sunshine, and giving an air of life and beauty to the grandeur of nature's habitations.

To the living, the expenditure of this sum means creating and perpetuating diseases the most virulent and malignant; the choking of our atmosphere in many instances with gases the most dangerous to health and to life itself; the perpetuation of memories the most ghastly and revolting to sensitive natures; for such never fail to make themselves a part of that vaunted sentiment surrounding the Christian tomb. It means hunger, want and despair, oftener than the world cares to investigate, to many of the poor survivors, whose last savings have been swept away—aye, and heavy debts contracted in order to afford the deceased member of the impoverished family a "decent, Christian burial."

CONCLUSIONS.

I. That the present system of burial is attended with danger to the living.

II. That abuses of the system are frequent, and at all times difficult to prevent, and sometimes become so serious as to result in overwhelming whole communities with pestilence and death.

III. That a residence near crowded grave-yards predisposes to diseases characterized by evidences of slow poisoning.

IV. That the system of burial, as now practiced, is necessarily attended with enormous expense, which is an oppressive drain on the resources of the poor.

V. That the general adoption of cremation would dispose of the dead rapidly at a nominal expense, compared with inhumation, and with entire sanitary safety to the living.

VI. That a similar disposal of the decaying organic products of large cities would tend to diminish the whole class of zymotic diseases which at present is the cause of considerably more than one-fourth of our death-rate.

